# Tissue-specific metal accumulation and tuber metabolic reprogramming in *Cyperus esculentus* under multiple-metal stress

**DOI:** 10.3389/fpls.2026.1849237

**Published:** 2026-08-28

**Authors:** Changsong Ren, Wenqi Xiao, Yijie Zhang, Haibo Wu, Jing Zhang, Bingliang Liu, Jun Li, Qiang Li, Xinhui Wang

**Affiliations:** 1 Institute of Agricultural Quality Standards and Detection Technology, Xinjiang Academy of Agricultural Sciences, Xinjiang, China; 2 College of Food and Biological Engineering, Chengdu University, Chengdu, China; 3 Xinjiang Academy of Agricultural Sciences, Xinjiang, China; 4 Xinjiang Academy of Agricultural Sciences, Turpan Agricultural Science Research Institute, Xinjiang, China; 5 Panzhihua University, Panzhihua, China

**Keywords:** *Cyperus esculentus*, heavy metal co-contamination, phytoremediation, transcriptomics, tuber

## Abstract

Understanding plant responses to co-occurring metal contamination is essential for evaluating their phytoremediation potential, yet the physiological and molecular responses of *Cyperus esculentus* (CES) to combined Cu, Zn, and Cd exposure remain poorly understood. Here, CES plants were exposed to combined Cu, Zn, and Cd stress at concentrations of 0–10 mg/L under hydroponic conditions, and plant growth, tissue-specific metal accumulation, physiological and biochemical responses, and tuber transcriptomic changes were systematically analyzed. Metal accumulation exhibited clear tissue specificity, with roots serving as the primary sites of metal retention, particularly for Cu, while culms accumulated considerable amounts of Zn and Cd and tubers showed comparatively lower but detectable metal accumulation. Increasing metal concentrations progressively inhibited plant growth and enhanced oxidative stress, as indicated by elevated H_2_O_2_ and malondialdehyde (MDA) levels, accompanied by stress-dependent adjustments in soluble sugars, reducing sugars, and flavonoids. In tubers, increased soluble sugar accumulation together with the downregulation of carbohydrate catabolism-related genes suggested a shift toward carbon reserve maintenance under metal stress. Under high-concentration exposure, tuber glutathione content increased nearly nine-fold, accompanied by sustained upregulation of a metallothionein-like protein gene and multiple stress-protective genes. These findings indicate that CES exhibits coordinated physiological and transcriptional responses to combined Cu/Zn/Cd stress and suggest that tubers may contribute to stress tolerance through carbon reserve adjustment and a potential glutathione-associated protective response. This study provides physiological and transcriptomic evidence for CES responses to Cu/Zn/Cd co-exposure and supports further evaluation of its phytoremediation potential in metal co-contaminated environments.

## 1 Introduction

Since the beginning of industrialization, human activities such as mining, agriculture, smelting, and industry have released metals from specific environments into adjacent ecosystems. Metals can be transported to distant ecosystems through air, soil, and river systems (Alloway, 2013; Kim et al., 2015). Elevated metal concentrations in agricultural land and rivers can lead to increased metal levels in crops, affecting growth and ultimately jeopardizing human health (Wei and Yang, 2010; Liu et al., 2018; Xiang et al., 2021). Many types of metals can be found in the same environment (Hou et al., 2019), and Cu, Zn, and Cd are the three most common metals in most areas (Yanqun et al., 2004; Zheng et al., 2007; Pietrzykowski et al., 2014). Previous studies have reported elevated metal concentrations in industrial soils, and the contents of Cu, Zn, and Cd can reach 29.7 mg/kg, 658 mg/kg, and 125.8 mg/kg, respectively (Jalali and Khanlari, 2008). In uncontaminated soils, the natural background levels are typically 20–30 mg/kg for Cu, 50–100 mg/kg for Zn and 0.1–0.5 mg/kg for Cd (Jalali and Khanlari, 2008; Alloway, 2013). In addition, these levels often exceed the toxicity thresholds for many plants, which are approximately 20–30 mg/kg for Cu, 100–200 mg/kg for Zn, and 5–10 mg/kg for Cd (Küpper and Andresen, 2016; Haider et al., 2021). Some studies have detected the metal contents in the capillary water of polluted soil, and the contents of Cu, Zn, and Cd can reach the highest levels of 6841 mg/kg, 39000 mg/kg, and 302 mg/kg, respectively (Lamb et al., 2009). These concentrations in contaminated water are orders of magnitude higher than those found in natural waters, where typical concentrations of copper are less than 0.01 mg/kg, those of zinc are 0.05 mg/kg, and those of cadmium are less than 0.001 mg/kg (Lamb et al., 2009; Alloway, 2013). However, total metal concentrations in soils, metal levels in soil pore water or aqueous environments, and plant-available metal fractions are not directly equivalent, because metal bioavailability is strongly influenced by metal speciation, adsorption to soil particles, organic matter, pH, and rhizosphere processes (Kim et al., 2015).

Plants have certain defenses to protect against metal uptake, transport, and homeostasis (Raza et al., 2022). When metal concentrations exceed homeostatic thresholds, plants activate mechanisms to maintain metal balance and reduce toxicity (Page and Feller, 2015; Tyagi et al., 2024). When the metal concentration in the environment is high, plants will create metal coordination compounds in the environment by secreting organic matter (Chen et al., 2017). Metal homeostasis is achieved within plant organs through the cell wall/membrane binding of metals; vesicle encapsulation of metals; the release of organic acids, polysaccharides, phytochelatins, and metallothioneins to chelate metals within the cell, and by regulating the activity of metal transporter proteins and transport pathways (Clemens et al., 2002; Peng and Gong, 2014; Emamverdian et al., 2015; Shahid et al., 2017). Plants also stabilize their cell structure by increasing the activity of proteins such as dehydrins, removing toxic metals (scavenging ROS) and regulating plant growth (Hara et al., 2003; Jozefczak et al., 2012).

CES is a perennial sedge in the family Cyperaceae. It forms edible underground tubers and can propagate through seeds, rhizomes, and tubers. The tubers are used as food and can be processed into beverages, flour, and oil, giving CES economic value as a tuber crop. CES has certain tolerance and accumulation capacity in metal-contaminated areas, especially in wastewater-contaminated environments (Chaturvedi et al., 2006; Yadav and Chandra, 2011). It can take up various metals, such as Fe, Mn, Cr, Zn, Cu, Ni, Pb, and Cd and accumulates most of them in the roots (Chandra and Yadav, 2011; Yadav and Chandra, 2011). Furthermore, while single-metal effects are well characterized, the combined effects of co-occurring metals are environmentally more relevant but remain poorly understood. In particular, the specific biochemical and genetic mechanisms underlying CES responses to mixed metal stress have not been elucidated. To address these gaps, we exposed CES to a range of concentrations (0, 0.5, 1, 2, 5, and 10 mg/L) of Cu, Zn, and Cd in hydroponic culture. This hydroponic design was used to establish a controlled gradient of bioavailable metal exposure and should not be interpreted as a direct simulation of total metal concentrations in field soils. Within this controlled framework, the selected concentrations were intended to represent environmentally relevant to moderately severe metal exposure levels, as preliminary experiments showed complete growth inhibition above 10 mg/L. We measured growth parameters, metal accumulation in different organs, physiological and biochemical indices, and performed transcriptomic analysis of tubers. This study provides a comprehensive physiological and molecular basis for understanding metal tolerance in CES and offers potential genetic resources for phytoremediation of multi-metal contaminated sites.

## 2 Materials and methods

### 2.1 Plants and culture conditions

For this study, CES tubers were obtained from a local commercial source in Baoding, Hebei Province, China. All tubers used in the experiment were selected from the same seed-tuber batch to reduce variation in planting material. To prepare them for cultivation, the tubers were cleaned and surface-sterilized by immersion in a 0.5% sodium hypochlorite solution for 30 minutes, followed by a thorough rinse with sterile purified water to remove any residual sterilant. Germination was induced by incubating the tubers in pure water within a plant incubator set to 30 °C, 70% relative humidity, and complete darkness (0 lx).

The hydroponic experiment was conducted at Chengdu University, Chengdu, China. After 60 h of germination, seedlings with emerging roots were transferred to a hydroponic system containing modified Hoagland nutrient solution. The nutrient solution contained 5.0 mM Ca(NO_3_)_2_·4H_2_O, 5.0 mM KNO_3_, 1.0 mM KH_2_PO_4_, 2.0 mM MgSO_4_·7H_2_O, 46 μM H_3_BO_3_, 9 μM MnCl_2_·4H_2_O, 0.8 μM ZnSO_4_·7H_2_O, 0.3 μM CuSO_4_·5H_2_O, 0.02 μM H_2_MoO_4_, and 50 μM Fe-EDTA. The initial pH of the nutrient solution was adjusted to 6.0 ± 0.1 using 0.1 M HCl or 0.1 M NaOH. The hydroponic solution was continuously aerated using an air pump to maintain dissolved oxygen availability during plant growth. The growth chamber simulated a diurnal cycle consisting of a 14-h light phase at 30 °C, 70% relative humidity, and 8000 lx, followed by a 10-h dark phase at 30 °C, 70% relative humidity, and 0 lx.

Plants were subjected to combined Cu, Zn, and Cd stress by adding CuSO_4_·5H_2_O, ZnSO_4_·7H_2_O, and CdCl_2_·2.5H_2_O to the nutrient solution. Six treatment groups were established with equal concentrations of the three metals: 0, 0.5, 1, 2, 5, and 10 mg/L (The corresponding molar concentrations were 7.87, 15.74, 31.47, 78.68, and 157.37 μM for Cu; 7.65, 15.30, 30.59, 76.48, and 152.95 μM for Zn; and 4.45, 8.90, 17.79, 44.48, and 88.96 μM for Cd.). These values refer to the concentration of each individual metal in the mixed solution, rather than the total concentration of the three metals. The nutrient solution was renewed every 3 days, and the corresponding concentrations of Cu, Zn, and Cd were re-added at each renewal to maintain the designed exposure levels throughout the 15-day treatment period. The solution pH was monitored during renewal and adjusted to 6.0 ± 0.1 when necessary. This concentration range was selected to evaluate responses to varying stress levels, as preliminary experiments showed that concentrations above 10 mg/L completely inhibited growth. Each treatment was conducted with three independent biological replicates (n=3), and each replicate contained nine uniformly sized tubers (Uniformity was controlled by selecting visually healthy tubers of similar size and without visible mechanical damage or disease symptoms. The tubers used in this experiment were stored seed tubers before germination.). After a 15-day treatment period, the plants were harvested, rinsed with saline solution (0.9% NaCl), and stored at -20 °C for subsequent analysis.

### 2.2 Determination of growth traits, physiological and biochemical indices, and metal content

Growth traits, including root length, plant height, and plant weight, were measured after treatment. Root length and plant height were measured using a root scanner (WinRHIZO, Canada) and WinRHIZO software (Version 2007d, Regent Instrument Inc., Canada). Plant fresh weight was measured for whole plants after treatment using an analytical balance (FA2204).

For metal quantification, culms, roots, and tubers were separated after harvest and rinsed thoroughly with 0.9% NaCl solution followed by ultrapure water to remove surface-adhered metal ions. The samples were oven-dried at 70 °C to constant weight, ground into fine powder, and stored in sealed tubes before digestion. Approximately 0.20 g of dried plant powder was accurately weighed into a microwave digestion vessel, followed by the addition of 6 mL concentrated HNO_3_ and 2 mL H_2_O_2_. The samples were pre-digested at room temperature for approximately 30 min and then digested using a microwave digestion system. The digestion program was set as follows: ramp to 120 °C within 5 min and hold for 5 min, then ramp to 180 °C within 10 min and hold for 20 min. After cooling to room temperature, the digested solutions were transferred to volumetric flasks and diluted to 25 mL with ultrapure water. The solutions were filtered through a 0.22 μm membrane before instrumental analysis.

The concentrations of Cu, Zn, and Cd in culms, roots, and tubers were determined using inductively coupled plasma optical emission spectroscopy (ICP-OES; Agilent 5110, USA), and the results were calculated and expressed as μg/g dry weight. Quality control was performed using reagent blanks, calibration standards, repeated measurements, and spike recovery tests. Reagent blanks were processed together with the samples to monitor potential contamination during digestion and measurement. Calibration curves were established using certified standard solutions of Cu, Zn, and Cd, and the correlation coefficients were maintained above 0.999. Selected samples were analyzed in triplicate to evaluate instrumental stability and analytical precision.

BCF and TF were calculated from replicate-level metal concentration data to evaluate metal accumulation and translocation capacity, and the detailed formulas and results are provided in **Supplementary Tables 1**, **2**.

### 2.3 Determination of indices

The contents or activities of several physiological and biochemical indices were measured in culms, roots, and tubers, including malondialdehyde (MDA), hydrogen peroxide (H_2_O_2_), protein, soluble sugar, reducing sugar, chlorophyll, flavonoids, glutathione, peroxidase (POD), and superoxide dismutase (SOD).

Physiological and biochemical indices were measured in culms, roots, and tubers using commercial assay kits (Nanjing Jiancheng Bioengineering Institute, Nanjing, China). The measured indices included malondialdehyde (MDA), hydrogen peroxide (H_2_O_2_), total protein, soluble sugar, reducing sugar, total chlorophyll, chlorophyll a, chlorophyll b, flavonoids, reduced glutathione (GSH), peroxidase (POD) activity, and superoxide dismutase (SOD) activity. Chlorophyll indices, including total chlorophyll, chlorophyll a, and chlorophyll b, were measured in culms. The corresponding assay kits were used according to the manufacturer’s instructions. Accession numbers have been added to the **Supplementary Table 3**.

### 2.4 RNA sequencing and data analysis

RNA-seq analysis of CES tubers under metal stress employed Illumina sequencing. Total RNA was extracted using TRIzol^®^ (Invitrogen, USA) with stringent quality control: RNA integrity number (RIN) > 8.0 (Agilent 2100 Bioanalyzer), A260/A280 = 1.8–2.0, and A260/A230 > 2.0 (NanoDrop spectrophotometer). Three biological replicates per treatment group underwent library preparation: (1) Poly(A)+ mRNA enrichment via oligo(dT) magnetic beads; (2) Random fragmentation using New England Biolabs (NEB) fragmentation buffer with divalent cations; (3) Library construction following NEB standard protocols (Parkhomchuk et al., 2009); (4) Library quantification via qRT-PCR (effective concentration > 2 nM). Qualified libraries were sequenced on Illumina platforms. Raw data were processed through: (a) Quality filtering (removal of adapters/low-quality bases) to generate clean reads; (b) *De novo* transcript assembly per sample using StringTie v2.1.7 (Pertea et al., 2015); (c) Integration of transcripts across all replicates into a unified transcript set.

For transcriptomic analysis, tuber samples from the 0, 1, and 5 mg/L treatments were selected and named CES_CK, CES_L, and CES_H, respectively. These groups represented the control, low-concentration, and high-concentration combined metal-stress treatments. The 2 mg/L treatment was considered an intermediate condition, whereas the 10 mg/L treatment caused severe injury and was therefore not selected for RNA-seq analysis. Correlation analysis and principal component analysis (PCA) were performed to evaluate replicate consistency and overall separation among treatment groups. Venn diagrams, clustered heatmaps, and volcano plots were used to visualize gene-expression differences among the treatment groups. GO and KEGG enrichment analyses of DEGs were performed using the clusterProfiler package. Enrichment results were ranked according to the adjusted P-value, and the top enriched GO terms and KEGG pathways were visualized.

### 2.5 Statistical analysis

Statistical analyses were performed to assess the magnitude of the differences between the samples. Differences among the six metal-treatment groups within the same organ or index were analyzed using one-way analysis of variance (ANOVA) followed by Tukey’s *post-hoc* test. Different lowercase letters in the figures indicate significant differences among treatments at P < 0.05. The letters were assigned according to the mean values, with “a” indicating the highest mean value, followed by “b”, “c”, and so on in descending order. Treatments sharing the same letter are not significantly different. The analysis was conducted using SPSS software (IBM SPSS Statistics 26, Chicago, USA), with significance set at p < 0.05. Graphical representations were generated with Origin software (Origin 2024, Northampton, USA). Gene expression levels were standardized using the fragments per kilobase of transcript per million mapped reads (FPKM) method, and differentially expressed genes (DEGs) were identified using DESeq2 software (|log2(FoldChange)| > 1.0 & padj ≤0.05). The padj was calculated by DESeq2 using the Benjamini-Hochberg procedure to control the false discovery rate (FDR).

## 3 Results

### 3.1 Plant growth

**Figure 1:**
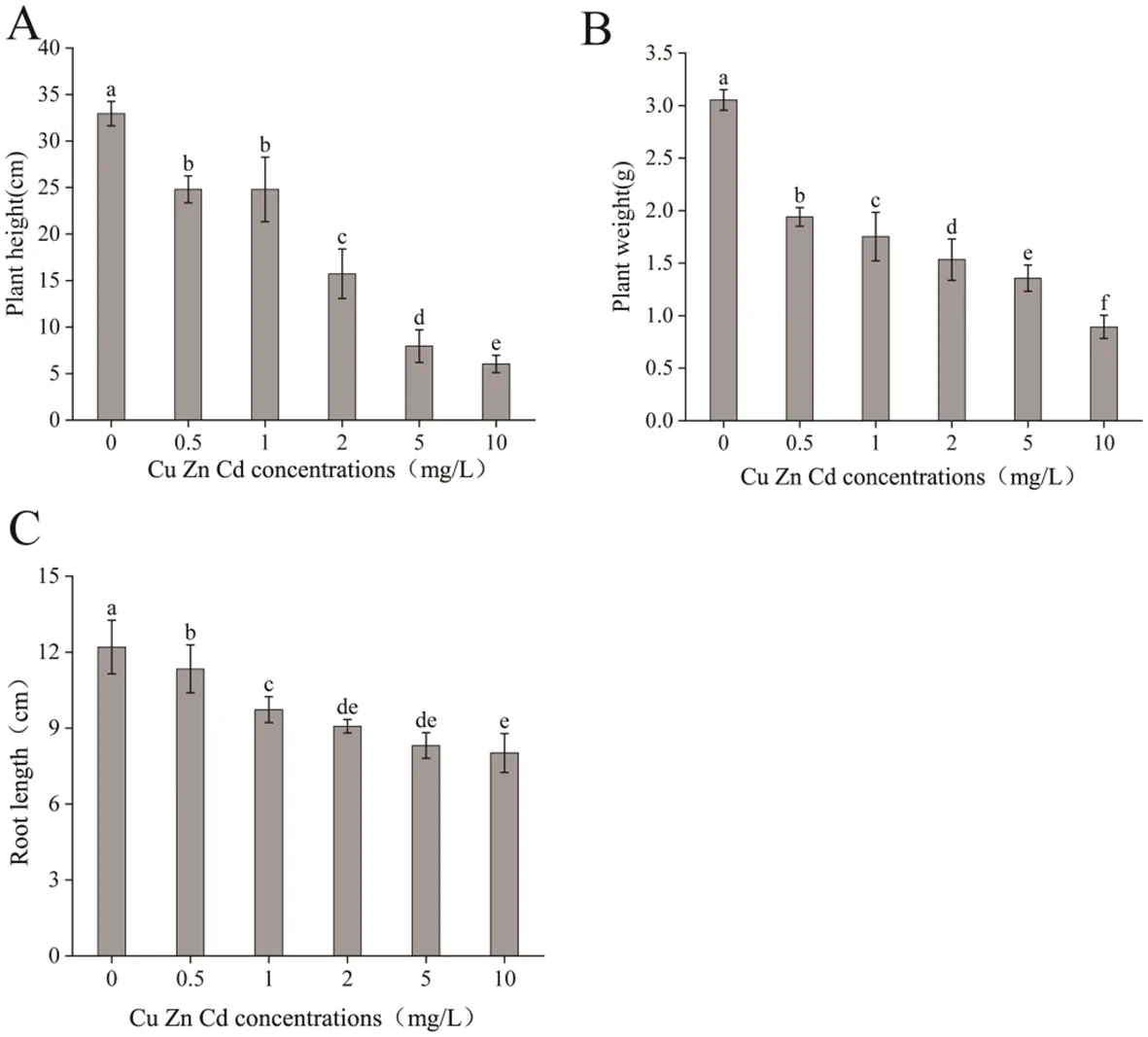
**(A-C)** Plant height, plant weight and root length of CES at different metal concentrations. Different letters indicate significant differences among treatments for the same growth trait at P < 0.05.

In the metal concentration range of 0–10 mg/L, the inhibitory effect on plant growth became stronger with increasing metal concentration. The symptoms included decreased plant height and yellow leaves, and the above-ground portions of the plants nearly withered at a concentration of 10 mg/L (**Supplementary Figure 1**). Significant decreases (*P* < 0.05) in plant height (**Figure 1A**), biomass (**Figure 1B**), and root length (**Figure 1C**) were detected with increasing metal concentrations.

### 3.2 Metal content

**Table 1** shows the metal contents in the Culms, roots, and tubers of the plants and the metal limits. Metal accumulation in Culms, roots, and tubers showed significant increases with treatment intensity: at 10 mg/L, Cu, Zn, and Cd contents reached 43.74, 147.45, 175.57 μg/g in Culms, 1497.26, 295.85, 409.18 μg/g in roots, and 10.05, 27.3, 11.81 μg/g in tubers. Compared with the control, Cu, Zn, and Cd contents increased markedly in all organs under high-concentration metal stress. Overall, roots served as the primary sites for metal retention, particularly for Cu, whereas culms also accumulated considerable amounts of Zn and Cd. Tubers showed lower metal accumulation than roots and culms but still exhibited significant increases in Cu, Zn, and Cd contents under high-concentration stress.

**Table 1:** Metal content in various organs of the CES (μg/g).

Metal concentration (mg/L)	Organ	Cu	Zn	Cd
0	Culm	8.42 ± 0.54e	12.96 ± 0.46f	5.47 ± 0.29e
	Root	9.10 ± 0.40e	9.75 ± 0.35d	6.52 ± 0.20d
	Tuber	1.17 ± 0.04f	10.71 ± 0.50d	1.20 ± 0.05e
0.5	Culm	13.46 ± 0.42d	19.68 ± 0.86e	19.51 ± 1.34d
	Root	58.34 ± 2.07e	18.87 ± 0.67d	22.17 ± 1.18d
	Tuber	1.93 ± 0.10e	12.10 ± 0.43cd	1.99 ± 0.09d
1	Culm	27.28 ± 1.28c	42.85 ± 1.52d	46.88 ± 1.47c
	Root	132.18 ± 4.68d	20.75 ± 1.10d	22.74 ± 0.81d
	Tuber	2.65 ± 0.12d	12.14 ± 0.43c	2.29 ± 0.11cd
2	Culm	37.24 ± 1.32b	64.06 ± 2.27c	55.50 ± 3.83c
	Root	414.99 ± 18.05c	133.36 ± 6.45c	153.18 ± 5.43c
	Tuber	3.56 ± 0.08c	12.83 ± 1.02c	2.62 ± 0.09c
5	Culm	39.61 ± 2.10b	97.03 ± 4.56b	116.08 ± 5.05b
	Root	778.03 ± 53.63b	218.92 ± 6.85b	274.10 ± 7.62b
	Tuber	4.22 ± 0.20b	17.89 ± 0.63b	4.67 ± 0.17b
10	Culm	43.74 ± 2.12a	147.45 ± 5.22a	175.57 ± 8.25a
	Root	1497.26 ± 46.87a	295.85 ± 13.91a	409.18 ± 21.71a
	Tuber	10.05 ± 0.36a	27.30 ± 0.97a	11.81 ± 0.63a

There was significant difference between the groups with different letters (P<0.05); For each metal element in different tissues, significance analysis was carried out independently.

To further evaluate the phytoremediation potential of CES, BCF and TF values were calculated based on replicate-level tissue metal concentrations and hydroponic exposure concentrations (**Supplementary Tables 1**, **2**). Root BCF values were generally higher than those of culms and tubers, especially for Cu, indicating that roots were the major metal-retention organs. Under the 10 mg/L treatment, root BCF values reached 149.73 ± 4.69 L/kg for Cu, 29.59 ± 1.39 L/kg for Zn, and 40.92 ± 2.17 L/kg for Cd. TF values from roots to tubers remained low across all treatments, suggesting limited metal translocation into tubers. However, Zn and Cd showed relatively higher translocation from roots to culms under low-concentration exposure. These results indicate that CES has strong root metal-retention capacity under combined Cu/Zn/Cd stress.

### 3.3 Changes in physiological and biochemical indices in plants

**Figure 2:**
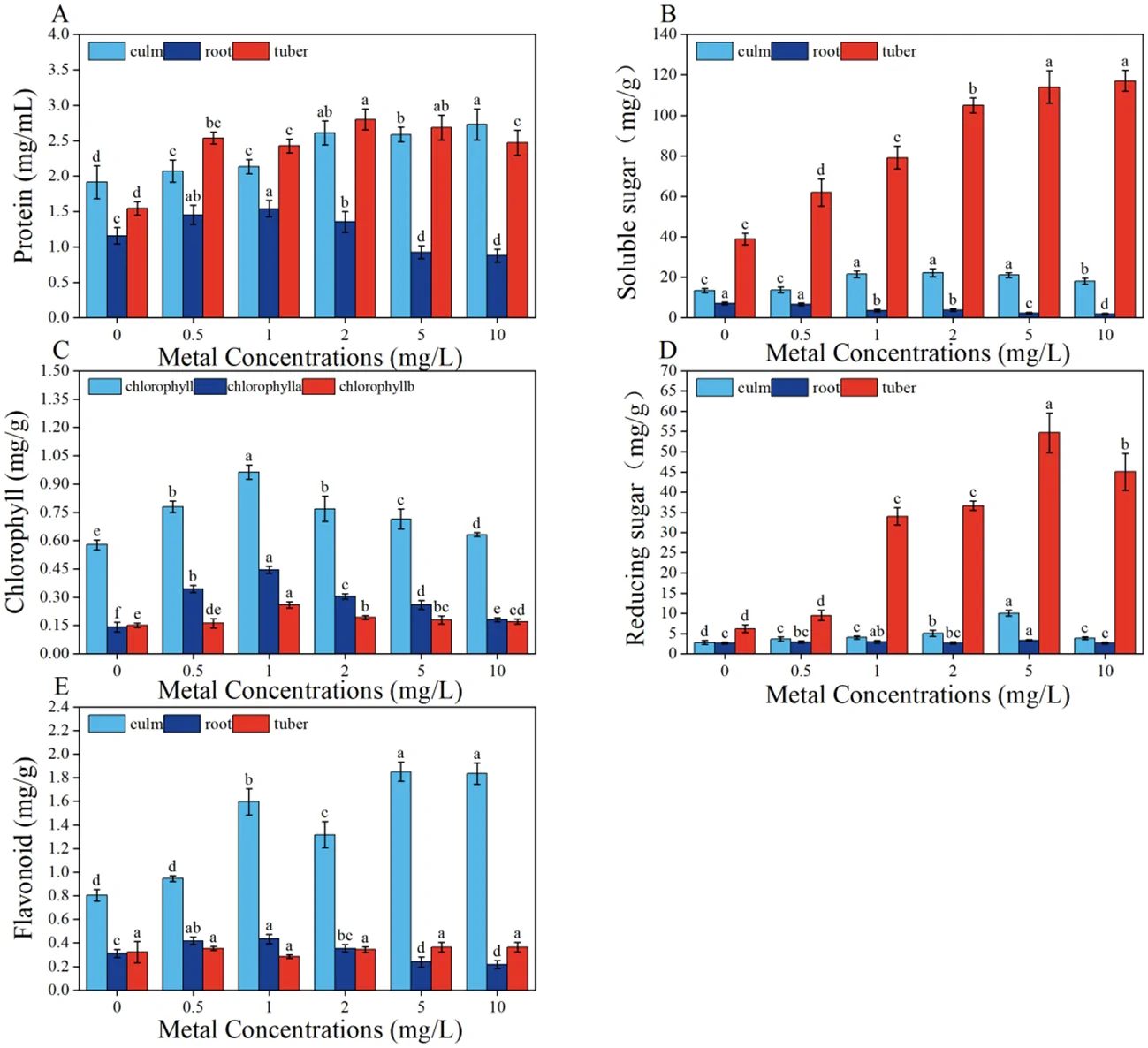
**(A-E) **Changes in the concentrations of various indices in three organs of CES at different concentrations. Different letters indicate significant differences among treatments within the same organ at *P* < 0.05.

Protein levels in Culms, roots, and tubers from the six treatment groups were assessed (**Figure 2A**). Protein content in roots and tubers initially increased, peaking at 1.54 mg/g (1 mg/L metal) in roots and 2.8 mg/g (2 mg/L) in tubers. Culm protein content peaked at 2.6 mg/g (2 mg/L). Soluble sugar in Culms (**Figure 2B**) first increased, peaking at 21.46 mg/g (1 mg/L), then decreased significantly at 10 mg/L (P < 0.05). In roots, soluble sugar declined with increasing metal concentration, dropping by 5.33 mg/g (accounting for approximately 76%) at 10 mg/L. Conversely, tuber soluble sugar increased, rising by 78.12 mg/g (accounting for approximately 201%) at 10 mg/L. Total chlorophyll, chlorophyll a, and b (**Figure 2C**) peaked at 1 mg/L (0.96, 0.44, and 0.26 mg/g, respectively) and declined thereafter. At 10 mg/L, reductions were 59.36%, 32.06%, and 34.31%, respectively, relative to peak values. Reducing sugar in Culms and tubers (**Figure 2D**) increased with metal concentration, peaking at 5 mg/L (10.02 mg/g and 54.67 mg/g, respectively). At 10 mg/L, reducing sugar contents decreased to 3.89 mg/g and 45.02 mg/g, respectively. Flavonoid content in Culms (**Figure 2E**) increased with metal concentration, peaking at 1.85 mg/g (5 mg/L). Root flavonoids peaked at 0.43 mg/g (1 mg/L) and gradually declined to 0.22 mg/g at 10 mg/L (accounting for approximately 70% of control).

**Figure 3:**
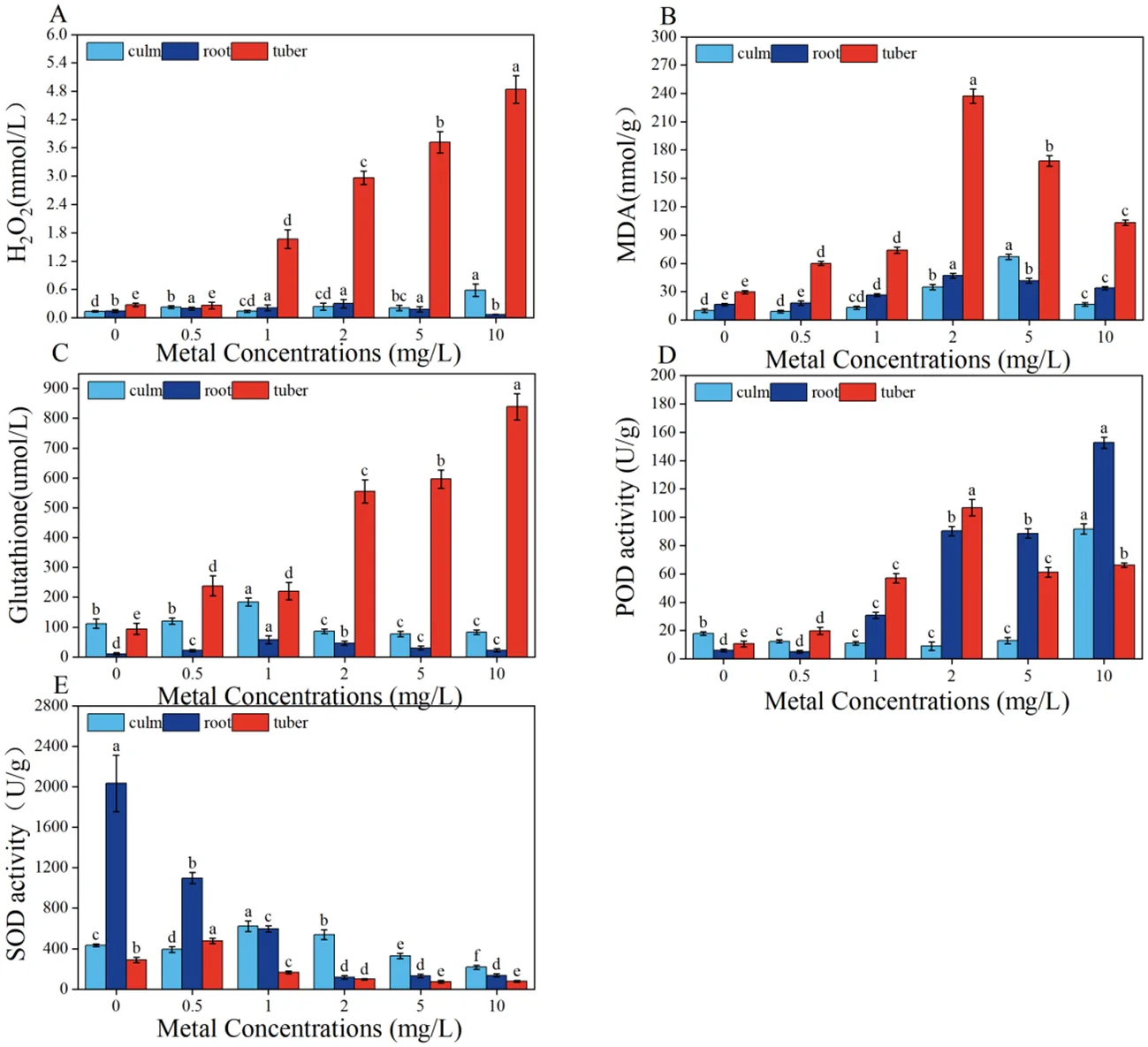
**(A-E) **Changes in oxidative stress-related indices and antioxidant enzyme activities in three CES organs under different metal concentrations. Different letters indicate significant differences among treatments within the same organ at *P* < 0.05.

H_2_O_2_ content in Culms and tubers (**Figure 3A**) increased with metal concentration, peaking at 10 mg/L (0.58 and 4.84 mmol/L), corresponding to increases of 432.14% and 1,761.40%, respectively. Root H_2_O_2_ rose from 0.14 mmol/L (0 mg/L) to a maximum of 0.30 mmol/L at 2 mg/L, then declined to 0.07 mmol/L at 10 mg/L. MDA content in Culms, roots, and tubers initially increased, then decreased after peaking (**Figure 3B**). Roots and tubers peaked at 2 mg/L (46.94 and 237.10 nmol/g), while Culms peaked at 5 mg/L (66.77 nmol/g), with increases of 285.29%, 803.28%, and 657.14%, respectively. Glutathione content in Culms and roots (**Figure 3C**) peaked at 1 mg/L (187.52 and 54.14 μmol/L), then declined to 83.33 and 22.62 μmol/L at 10 mg/L. In tubers, glutathione increased with metal concentration, reaching 845.23 μmol/L at 10 mg/L—an 898.73% increase. POD activity in Culms and roots (**Figure 3D**) correlated positively with metal concentration, reaching 91.70 and 152.59 U/g at 10 mg/L (increases of 511.57% and 2,512.20%). Tuber POD peaked at 2 mg/L (106.81 U/g), then declined to 61.33 U/g at 5 mg/L. SOD activity in Culms and tubers (**Figure 3E**) initially increased, then decreased. Culm SOD peaked at 1 mg/L (620.88 U/g) and fell to 216.21 U/g at 10 mg/L; tuber SOD peaked at 0.5 mg/L (477.98 U/g) and dropped to 76.17 U/g at 10 mg/L. SOD activity in the roots was negatively correlated with metal concentration, decreasing to 136.83 U/g at 10 mg/L, a drop of approximately 93.27%.

### 3.3 Metal-induced changes in gene expression in tubers

#### 3.3.1 Differential gene analysis

The changes in the above physiological and biochemical indicators indicate that CES responds to combined metal stress by regulating antioxidant-related metabolites and enzymes, including glutathione and POD, as well as osmotic regulatory metabolites such as soluble sugars and reducing sugars. We further analyzed tuber transcriptome data to investigate the relationship between gene expression dynamics and physiological responses.

**Figure 4:**
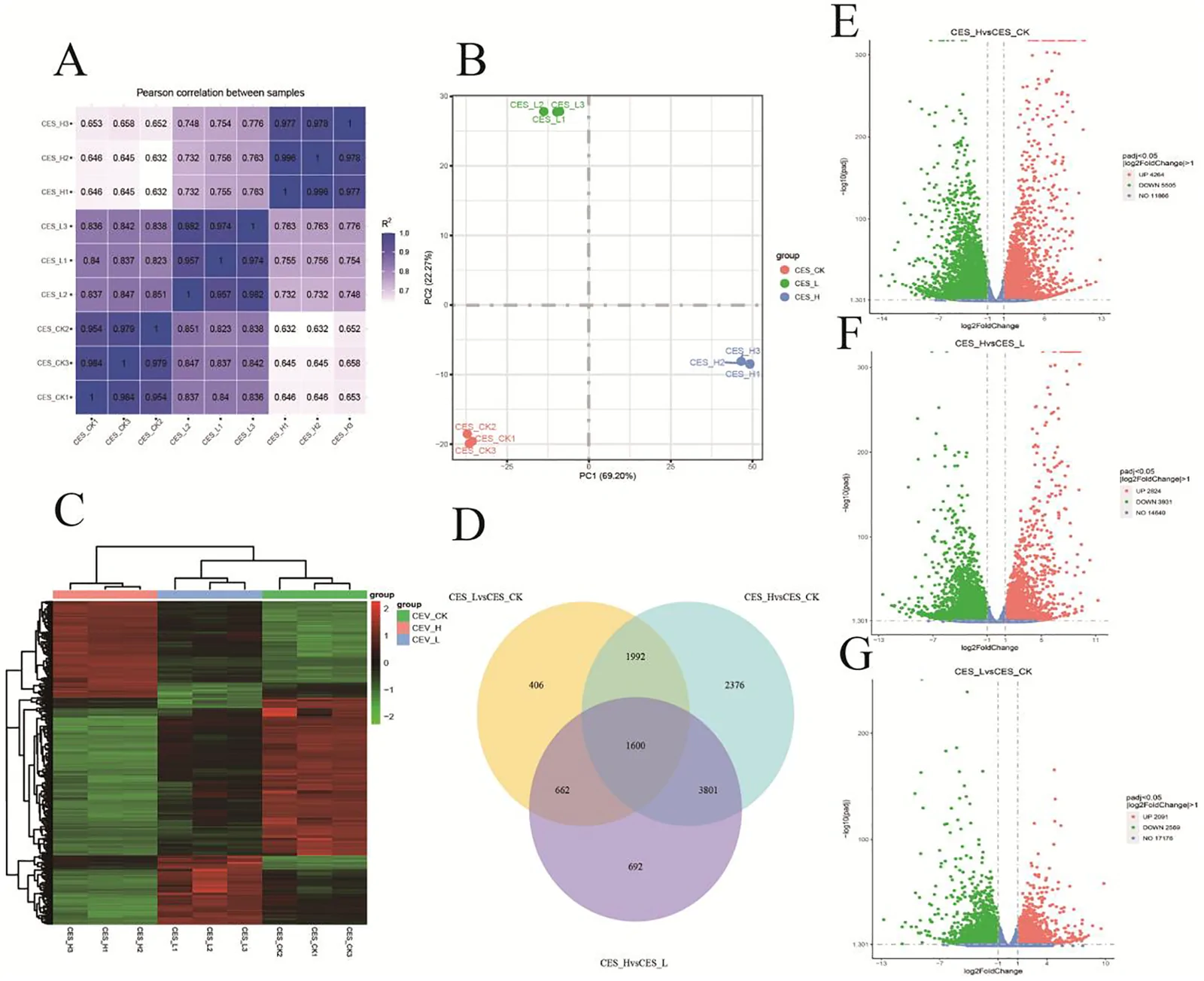
Correlation **(A)** and principal component analysis **(B)** of gene expression in three sample groups. Cluster heatmaps **(C)** and Venn plots **(D)** showing gene expression differences among three sample groups. **(E)**: CES_H VS CES_CK, **(F)** CES_H VS CES_L, **(G)** CES_LVS CES_CK. A VS B, A-experimental group, B-control group. The horizontal axis in the figure represents the expression fold change of genes in the treatment and control groups (log2FoldChange), and the vertical axis represents the significant level of gene expression difference in the treatment and control groups (- log10padj or - log10pvalue). The dashed line represents the threshold line for screening differentially expressed genes, with upregulated genes represented by red dots and downregulated genes represented by green dots.

Sample-correlation values were high among biological replicates within each treatment (0.954–0.996) and lower between treatments (0.632–0.851). PCA separated CES_CK, CES_L, and CES_H into three distinct clusters, with PC1 and PC2 accounting for 69.20% and 22.27% of the total variance, respectively (**Figures 4A, B**). These results demonstrate good biological-replicate consistency and distinguishable overall transcriptional states among the three selected treatment groups. Compared with those in the control group, there were 9,769 DEGs in the high concentration treatment group (**Figure 4E**), and 4,660 DEGs in the low-concentration treatment group (**Figure 4G**). There were 6,755 genes whose expression significantly differed between the low concentration and high concentration metal treatment groups (**Figure 4F**).

**Figure 5:**
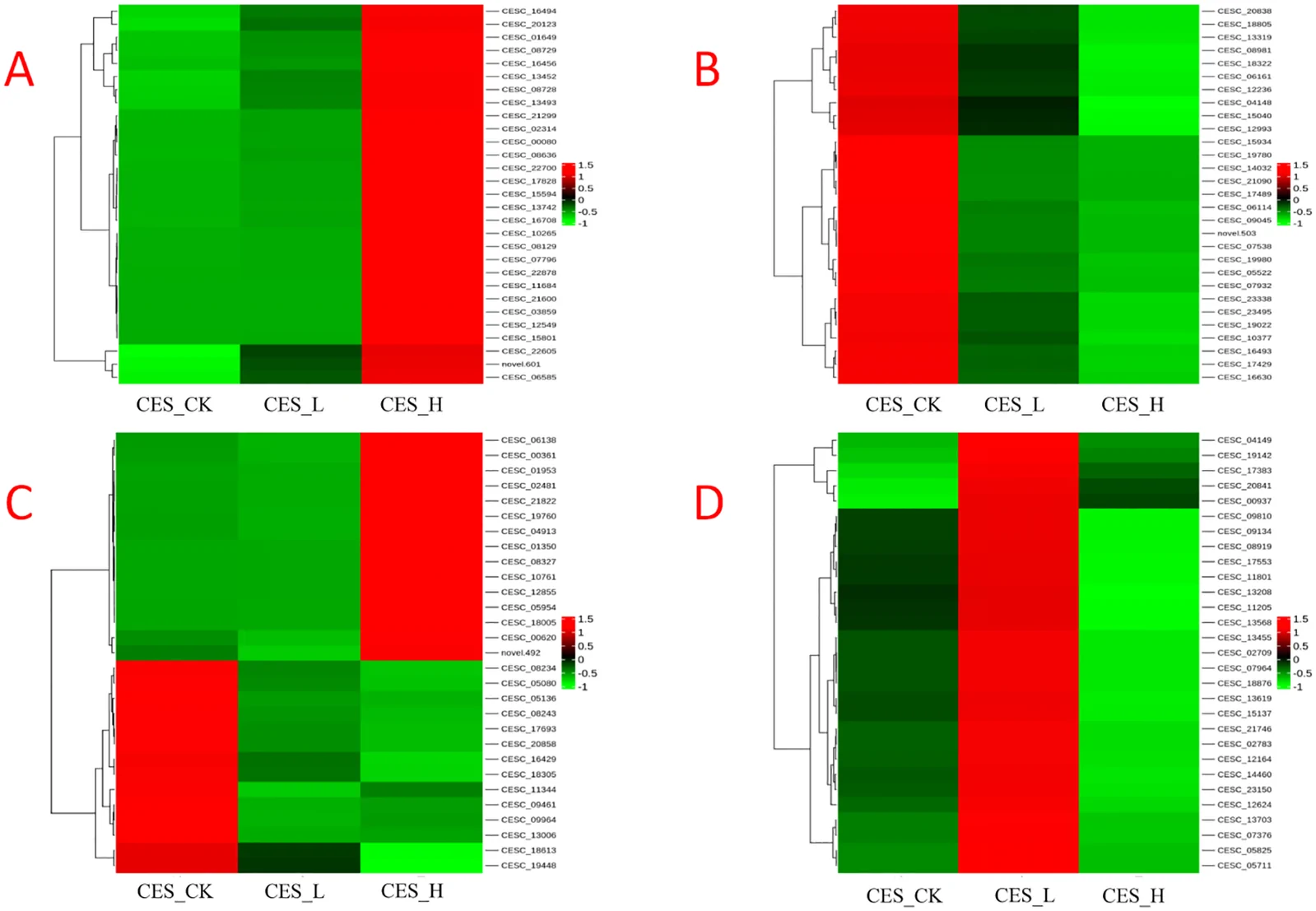
Changes in gene expression levels in tubers treated with different concentrations of metal. For clarity, each panel displays the 30 genes showing the largest absolute expression differences between CES_CK and CES_H within the corresponding category. **(A)** Continuously rising genes, **(B)** Continuously downregulated genes, **(C)** First downregulated and then upregulated genes, **(D)** Upregulation followed by downregulation of genes.

A total of 2,264 genes showed clear concentration-dependent expression changes. Among these genes, 406 were continuously upregulated with increasing metal concentration (**Figure 5A**). These genes are related to stress proteins, heat shock proteins, metallothionein, membrane proteins, pigments, oxidases, and transferases. These include Metallothionein-like protein type 2 (CESC_16708), Seed dormancy control (CESC_21299), Cytochrome P450 71D55 (CESC_08729), and Late embryogenesis abundant protein D-34 (CESC_03859), among others. Another 727 genes were continuously downregulated as metal concentration increased (**Figure 5B**). These genes are mostly linked to the transport, breakdown, and processing of carbohydrates, amino acids, pigments, and phosphates. These genes include Metallothionein type 2b (CESC_06114), Probable aquaporin PIP2-2 (CESC_05080), Alpha-galactosidase (CESC_17489), Beta-fructofuranosidase 1 (CESC_19780), MYB-related transcription factor LHY (CESC_19980), and Dof zinc finger protein DOF3.6 (CESC_15040), among others.

We also noticed two other groups of genes with special patterns. The first group had 377 genes. Their expression levels went down during low-concentration metal treatment, but then went up again during high-concentration treatment (**Figure 5C**). Among these, 67 genes showed higher expression levels in the high-concentration group than in the control group. These genes are involved in peptides, organic acids, cytokinins, vacuoles, pigments, membrane transporters, cation transporters, and metal ion exchange proteins. Examples of genes include Cytokinin dehydrogenase 6 (CESC_01350), Vacuolar protein sorting-associated protein 62 (CESC_01953), Dehydrin (CESC_17693), and NADP-dependent malic enzyme (CESC_08481).

The second group had 754 genes. Their expression levels went up during low-concentration treatment, but went down during high-concentration treatment (**Figure 5D**). For 272 of these genes, their expression levels in the high-concentration group were even lower than those in the no-metal group. These genes are involved in functions related to metal transfer proteins, polysaccharide hydrolases, protein ligases, glycosyltransferases, oxidases, amino acid transporters, chlorophyll binding proteins, and organic acid transporters. Examples of genes include Ribulose bisphosphate carboxylase small chain, chloroplastic (CESC_20841), Phosphoenolpyruvate carboxykinase [ATP] (CESC_05711), Chlorophyll a-b binding protein, chloroplastic (CESC_11767), and Copper transport protein ATX1 (CESC_13208).

#### 3.3.2 GO functional enrichment

**Figure 6:**
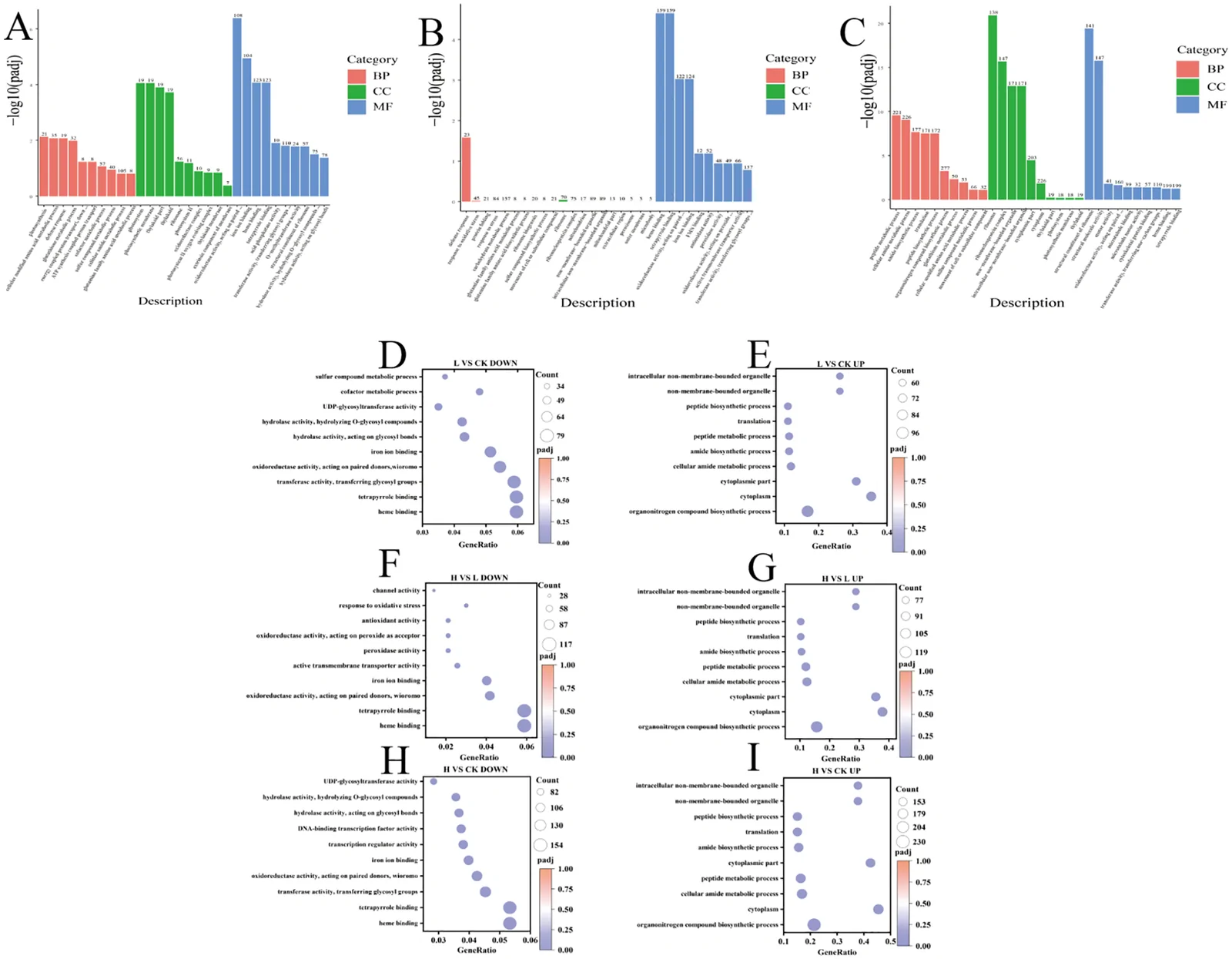
GO enrichment analysis histogram **(A)** CES_L vs. CES_CK, **(B)** CES_H vs. CES_L, and **(C)** CES_H vs. CES_CK. Bar plots show the top 30 enriched GO terms. Bubble plots show the top 10 enriched GO terms for downregulated and upregulated genes: **(D)** CES_L vs. CES_CK downregulated genes, **(E)** CES_L vs. CES_CK upregulated genes, **(F)** CES_H vs. CES_L downregulated genes, **(G)** CES_H vs. CES_L upregulated genes, **(H)** CES_H vs. CES_CK downregulated genes, and **(I)** CES_H vs. CES_CK upregulated genes.

The low-concentration group was first compared with the control group. Metal stress significantly changed biological processes, cellular components, and molecular functions in CES tubers. Some genes were downregulated (**Figure 6D**). These genes are linked to tetrapyrrole binding, iron ion binding, glucose metabolism, defense response, heme binding, and cofactor metabolism. Other genes were upregulated (**Figure 6E**). These are involved in amide biosynthesis, organic compound biosynthesis, translation, peptide biosynthesis, cellular amide metabolism, transition metal ion transport, metal cluster binding, photosystem activity, ion transport, photosynthesis, and cation transport.

The high-concentration group was then compared with the low-concentration group. Clear differences were found in gene functions. Some genes were strongly downregulated (**Figure 6F**). They are linked to oxidative stress, heme binding, tetrapyrrole binding, membrane transport, and metal ion binding. Other genes were upregulated (**Figure 6G**). These are related to amides, peptides, organic nitrogen, organic acids, amino acids, cytoplasm, and binding proteins. Some functions, such as those related to ribosome structure, kept increasing as metal concentration went up.

The high-concentration group was compared with the control group. Many genes tied to defense, heme binding, tetrapyrrole binding, oxidoreductase activity, and iron ion binding showed clear changes. Some genes were downregulated (**Figure 6H**). These are linked to heme binding, tetrapyrrole binding, transferase activity, glycosyl transfer, organic nitrogen compound synthesis, cytoplasm, and cellular amide metabolism. Other genes with related roles were upregulated (**Figure 6I**).

#### 3.3.3 KEGG functional enrichment

**Figure 7:**
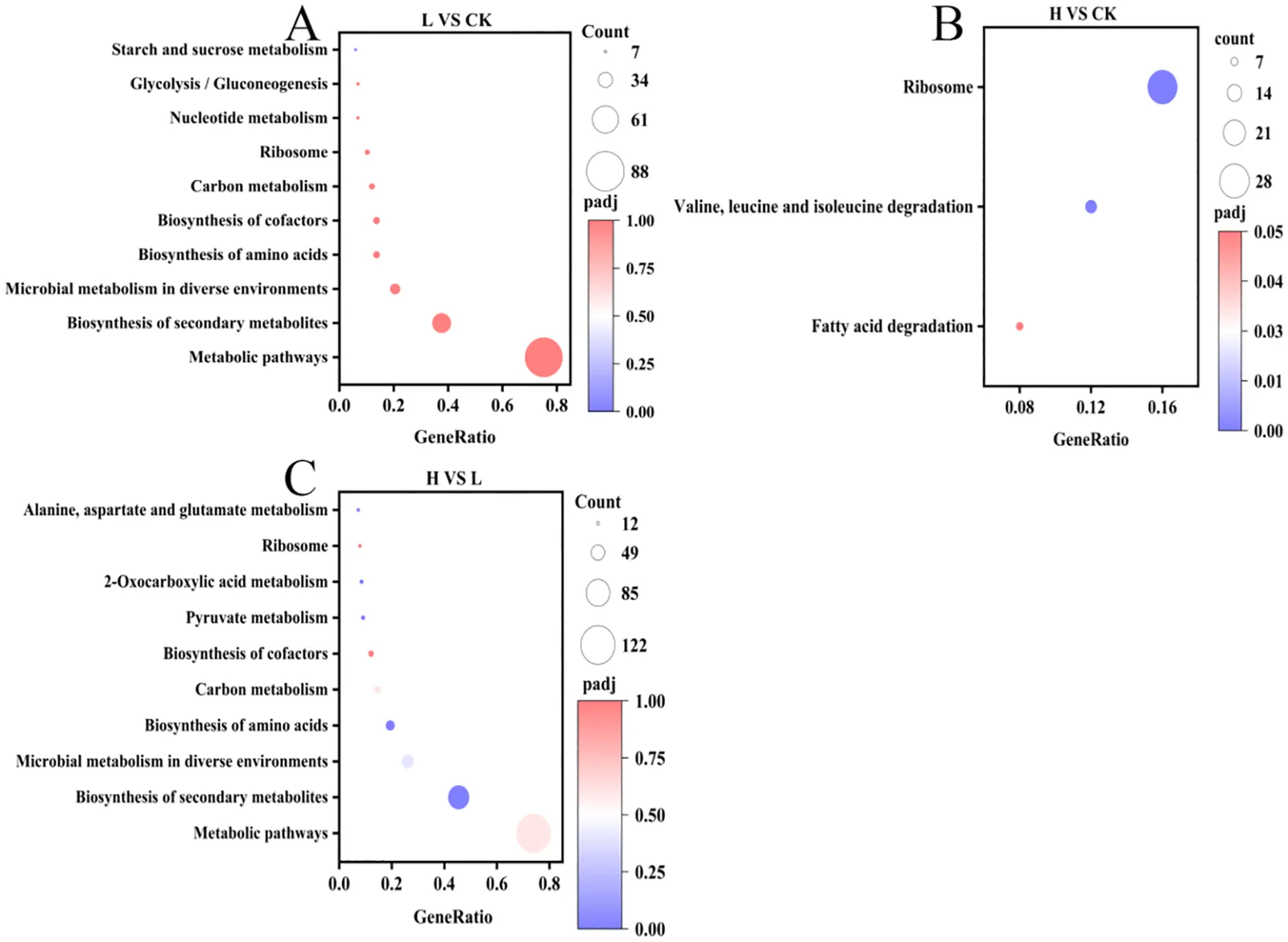
KEGG pathway enrichment analysis of DEGs under different metal treatments. Only the top 10 enriched pathways are shown; pathways with fewer than 10 enriched genes are also included. **(A)** CES_L vs. CES_CK, **(B)** CES_H vs. CES_CK, and **(C)** CES_H vs. CES_L.

KEGG pathway enrichment analysis revealed that, compared to those in the control sample, many genes whose expression significantly differed were enriched in metabolic pathways, biosynthesis of secondary metabolites, microbial metabolism in diverse environments, biosynthesis of amino acids, and functional pathways such as ribosomes. However, the degree of enrichment did not reach a significant level (**Figure 7A**).

KEGG pathway enrichment analysis revealed that, compared with the control sample, the high-concentration metal treatment group was significantly enriched in the valine, leucine, and isoleucine degradation and fatty acid degradation functional pathways. The genes involved in these pathways were all significantly downregulated (**Figure 7B**).

Compared with those in the samples subjected to low-concentration metal treatment, significantly different genes in the samples subjected to high-concentration metal treatment were enriched in functional pathways such as metabolic pathways; biosynthesis of secondary metabolites; microbial metabolites in diverse environments; biosynthesis of amino acids; ribosomes; fatty acid metabolism; and valine, leucine, and isoleucine degradation, but the degree of enrichment did not reach a significant level (**Figure 7C**).

## 4 Discussion

Given that tubers are the storage organs of CES and showed clear metabolic changes under combined metal stress, the Discussion mainly focuses on tuber carbon metabolism and glutathione-associated stress responses. Under combined metal stress, CES tubers showed marked metabolic adjustment rather than a confirmed active regulatory strategy. Under high-concentration metal treatments, the soluble sugar content in tubers increased by more than 200% compared with the control group (*P* < 0.05, **Figure 2B**). This substantial accumulation suggests that tubers serve as carbon storage organs. Concurrently, reducing sugar levels in tubers continued to rise, peaking at the 5 mg/L treatment (*P* < 0.05, **Figure 2D**). Beyond serving as essential energy sources, sugars also play critical roles under stress conditions by acting as metabolic signals, thereby maintaining cellular structure, photosynthetic capacity, and reactive oxygen species detoxification (Ahmad et al., 2020). As metal concentrations increased, genes involved in sugar catabolism, such as α-galactosidase (CESC_17489) and fructosidase (CESC_19780), were continuously downregulated (**Figure 5B**). Conversely, genes associated with carbon fixation and sugar synthesis, including ribulose bisphosphate carboxylase small chain (CESC_20841) and phosphoenolpyruvate carboxykinase (CESC_05711), were upregulated under moderate metal treatments (**Figure 5D**). In addition, a candidate SnRK1-related regulatory subunit gene, CESC_07904, was markedly upregulated under high-concentration metal stress, suggesting that sugar/energy signaling may participate in tuber metabolic adjustment. At the pathway level, KEGG analysis showed that all DEGs associated with valine, leucine, and isoleucine degradation and fatty acid degradation were downregulated in CES_H versus CES_CK. Because branched-chain amino acid and fatty acid catabolism can provide alternative respiratory substrates and carbon intermediates that feed the TCA cycle and support ATP production (Le and Millar, 2023), their coordinated downregulation, together with reduced sugar catabolism, is consistent with decreased consumption of metabolic reserves under metal stress, although general metabolic inhibition cannot be excluded. Together, these correlative physiological and transcriptomic results suggest a possible carbon-conservation response in tubers, involving sugar accumulation, reduced expression of sugar catabolism-related genes, and altered sugar/energy signaling. However, this interpretation requires further validation through targeted carbon flux and metabolic analyses.

Under combined metal stress, oxidative stress responses differed among plant organs. In culms and tubers, H_2_O_2_ content increased markedly with increasing metal concentration (*P* < 0.05, **Figure 3A**), indicating enhanced ROS accumulation under severe metal stress. By contrast, root H_2_O_2_ content showed a non-linear response, increasing from the control to the 2 mg/L treatment and then declining at 10 mg/L. This decrease should not be interpreted simply as reduced oxidative stress. Given the strong inhibition of root growth and the substantial metal accumulation in roots under high-concentration treatment, the lower H_2_O_2_ level at 10 mg/L may reflect severe root injury, reduced metabolic activity, altered ROS generation, or enhanced ROS-scavenging processes under extreme stress. Further root-specific analyses, such as root viability staining, ROS localization, and antioxidant enzyme assays, are needed to clarify this response.

Under high-concentration metal stress, the glutathione content in tubers increased nearly nine-fold compared with the control group (*P* < 0.05, **Figure 3C**). Concurrently, the levels of hydrogen peroxide and malondialdehyde rose sharply, indicating severe oxidative damage (*P* < 0.05, **Figures 3A, B**). Despite this severe oxidative stress, the marked accumulation of glutathione in tubers is consistent with an enhanced glutathione-associated response, although it does not by itself demonstrate increased glutathione biosynthetic capacity. Glutathione is a central component of plant responses to heavy metal stress (Jozefczak et al., 2012), and regulates the intracellular utilization of sulfur to support protein synthesis and stress responses (Rouhier et al., 2008). However, the candidate glutathione synthetase gene CESC_04008, annotated as a GSH2-like gene, was downregulated under high-concentration metal stress. The divergence between GSH accumulation and CESC_04008 expression indicates that transcript abundance alone may not fully capture the regulation of the GSH pool. Information on the activities of γ-glutamylcysteine ligase and glutathione synthetase, together with the availability of the precursors cysteine and glutamate, would help determine whether enzyme-level regulation and precursor supply contribute to the observed GSH accumulation.

In this study, a metallothionein-like protein gene (CESC_16708), which may function together with glutathione-related detoxification processes, was continuously upregulated (**Figure 5A**). Metallothioneins are essential elements for maintaining metal homeostasis in plant cells (Kumar et al., 2022). Glutathione is also involved in stress protection and development-related signaling processes in plant cells (Considine and Foyer, 2021; Iqbal et al., 2024). Stress protection genes, such as late embryogenesis abundant protein D-34 (CESC_03859) and seed dormancy control protein (CESC_21299), were consistently upregulated (**Figure 5A**). Overexpression of late embryogenesis abundant proteins increases plant tolerance to metal stress (Liu et al., 2019). Genes associated with desiccation protection and metabolic regulation, such as dehydrin (CESC_17693) and NADP-dependent malic enzyme (CESC_08481), were strongly upregulated under high metal treatments (**Figure 5C**). Dehydrins have also been found to scavenge ROS and bind metal ions in plant cells (Riyazuddin et al., 2022). Synergistic interactions exist among various stress-protective elements; for example, dehydrins also enhance the activities of peroxidase and glutathione transferase in plants (Liu et al., 2015; Nguyen et al., 2020). Genes associated with membrane and ion transport also responded to combined metal stress. For example, probable aquaporin PIP2-2 (CESC_05080) was continuously downregulated, while a gene annotated as copper transport protein ATX1 (CESC_13208) showed a low-concentration induction followed by repression under high-concentration treatment (**Figures 5B, D**). These patterns suggest that CES tubers may adjust water and ion transport processes under increasing multi-metal stress, although the specific roles of these transport-related genes remain to be validated. Collectively, these correlative physiological and transcriptomic results suggest that CES tubers may activate a glutathione-associated protective response under high-concentration metal stress. We propose that increased glutathione accumulation, together with the upregulation of metallothionein-like and stress-protective genes, may contribute to the mitigation of metal-induced oxidative damage. This interpretation requires further validation through analyses of glutathione biosynthetic enzyme activities, precursor pools, and GSH turnover rates, together with functional experiments.

Beyond tuber metabolic responses, the phytoremediation relevance of CES can also be understood by comparison with well-established wetland macrophytes. Previous studies have shown that *Phragmites australis* and *Typha angustifolia* are widely investigated for the phytoremediation of multi-metal-contaminated water and wetlands (Chandra and Yadav, 2011). In mixed-metal systems, *Phragmites* has been reported to accumulate several metals in aboveground tissues (Milke et al., 2020), whereas *Typha* often shows stronger root-retention characteristics for metals such as Cu, Cd, Cr, Pb, and Fe (Bah et al., 2011). Consistent with these reports, the present study showed that CES retained large amounts of Cu, Zn, and Cd in roots, particularly Cu, while translocation to tubers remained limited. The calculated BCF and TF values further support this pattern, indicating strong root metal-retention capacity and restricted movement of metals into storage organs. Therefore, CES may be more suitable for phytostabilization or rhizofiltration-type applications, in which metals are mainly retained in roots or removed from aqueous systems. Compared with previous studies that mainly evaluated metal accumulation in wetland macrophytes, the present study further provides physiological and transcriptomic evidence for tuber metabolic adjustment under multi-metal stress, thereby expanding our understanding of how CES storage organs respond to metal co-contamination. It should be noted that when utilizing CES for phytoremediation in contaminated environments, the harvested biomass should be distinguished from biomass intended for food or feed and managed as remediation biomass.

Nevertheless, the current findings are primarily derived from physiological measurements, metabolite profiling, and transcriptomic analysis, which provide correlative evidence rather than direct causal proof. Furthermore, the RNA-seq results were not independently validated via qRT-PCR. Consequently, the expression patterns of key candidate genes—including CESC_16708, CESC_03859, CESC_21299, CESC_05080, and CESC_19780—involved in metal detoxification, stress protection, membrane transport, and carbohydrate metabolism should be interpreted with caution and further validated using qRT-PCR in future studies. Therefore, the proposed glutathione-associated antioxidant defense model should be interpreted as a working hypothesis. Because samples were collected only after 15 days of treatment, the current results represent endpoint responses and cannot distinguish early stress responses from long-term adaptive processes. Future work should employ qRT-PCR validation, time-course sampling, genetic manipulation, and biochemical assays to dissect the hierarchical regulation and functional interplay of these stress-responsive components. In addition, transcriptomic profiling across a broader and more continuous concentration gradient, combined with comparative analyses of roots, culms, and tubers, would further clarify concentration-dependent and organ-specific molecular responses. Metal bioavailability in soils is strongly affected by soil pH, organic matter, mineral adsorption, metal speciation, microbial activity, and rhizosphere processes, which may alter metal uptake and toxicity compared with hydroponic exposure (Kim et al., 2015). Therefore, at a broader experimental scale, future studies should extend the present controlled hydroponic framework to long-term soil-based and field experiments spanning complete growth cycles. Field-based assessments of biomass production, metal stabilization and removal efficiency, and the consistency of BCF, TF, and root-retention patterns would then provide a stronger basis for evaluating the practical applicability of CES in long-term phytoremediation.

## 5 Conclusion

This study reveals that CES responds to combined Cu, Zn, and Cd stress through integrated physiological and transcriptional adjustments. Metal accumulation was tissue-specific, with roots acting as the main sites of metal retention, especially for Cu, while culms accumulated considerable amounts of Zn and Cd and tubers exhibited lower but significant metal uptake. Increasing metal concentrations inhibited growth and induced oxidative stress, but tubers showed a possible carbon-conservation response, as reflected by soluble sugar accumulation and downregulation of catabolism-related genes. Glutathione content in tubers increased nearly nine-fold under high stress, accompanied by sustained upregulation of a metallothionein-like protein gene and multiple stress-protective genes. These results support a proposed model in which CES tubers may coordinate glutathione-associated antioxidant defense and carbon reserve management to alleviate metal-induced damage. These findings provide preliminary physiological and transcriptomic evidence for multi-metal stress responses in CES and support further evaluation of its phytoremediation potential in co-contaminated environments.

## Data Availability

The data presented in the study are deposited in the NCBI Sequence Read Archive (SRA) repository, accession number PRJNA1515122.
