# Supplementary material for: Tissue-specific metal accumulation and tuber metabolic reprogramming in Cyperus esculentus under multiple-metal stress
Source: Front Plant Sci. 2026 Aug 28;17:1849237. doi: 10.3389/fpls.2026.1849237 (PMC13561833; doi:10.3389/fpls.2026.1849237)
Supplement: Supplementary file 1 [file SupplementaryFile1.docx]

Fig. S1: Representative photographs of hydroponically grown Cyperus esculentus plants under control and combined Cu, Zn, and Cd treatment conditions.

| **Treatment (mg/L)** | **Organ** | **Cu BCF** | **Zn BCF** | **Cd BCF** |
| --- | --- | --- | --- | --- |
| 0.5 | Culm | 26.92 ± 0.84 | 39.36 ± 1.71 | 39.02 ± 2.69 |
|  | Root | 116.68 ± 4.13 | 37.74 ± 1.34 | 44.33 ± 2.35 |
|  | Tuber | 3.85 ± 0.20 | 24.19 ± 0.86 | 3.98 ± 0.19 |
| 1 | Culm | 27.28 ± 1.28 | 42.85 ± 1.52 | 46.88 ± 1.47 |
|  | Root | 132.18 ± 4.68 | 20.75 ± 1.10 | 22.74 ± 0.81 |
|  | Tuber | 2.65 ± 0.12 | 12.14 ± 0.43 | 2.29 ± 0.11 |
| 2 | Culm | 18.62 ± 0.66 | 32.03 ± 1.13 | 27.75 ± 1.91 |
|  | Root | 207.50 ± 9.03 | 66.68 ± 3.22 | 76.59 ± 2.71 |
|  | Tuber | 1.78 ± 0.04 | 6.42 ± 0.51 | 1.31 ± 0.05 |
| 5 | Culm | 7.92 ± 0.42 | 19.41 ± 0.91 | 23.22 ± 1.01 |
|  | Root | 155.61 ± 10.73 | 43.78 ± 1.37 | 54.82 ± 1.52 |
|  | Tuber | 0.84 ± 0.04 | 3.58 ± 0.13 | 0.93 ± 0.03 |
| 10 | Culm | 4.37 ± 0.21 | 14.75 ± 0.52 | 17.56 ± 0.83 |
|  | Root | 149.73 ± 4.69 | 29.59 ± 1.39 | 40.92 ± 2.17 |
|  | Tuber | 1.01 ± 0.04 | 2.73 ± 0.10 | 1.18 ± 0.06 |

Table S1:BCF values of Cu, Zn, and Cd in CES organs under combined metal stress

| **Treatment (mg/L)** | **TF type** | **Cu TF** | **Zn TF** | **Cd TF** |
| --- | --- | --- | --- | --- |
| 0.5 | Culm/Root | 0.231 ± 0.008 | 1.044 ± 0.075 | 0.884 ± 0.106 |
|  | Tuber/Root | 0.033 ± 0.001 | 0.642 ± 0.045 | 0.090 ± 0.001 |
| 1 | Culm/Root | 0.206 ± 0.003 | 2.071 ± 0.178 | 2.063 ± 0.075 |
|  | Tuber/Root | 0.020 ± 0.002 | 0.586 ± 0.011 | 0.101 ± 0.008 |
| 2 | Culm/Root | 0.090 ± 0.003 | 0.481 ± 0.019 | 0.363 ± 0.037 |
|  | Tuber/Root | 0.009 ± 0.001 | 0.096 ± 0.006 | 0.017 ± 0.001 |
| 5 | Culm/Root | 0.051 ± 0.002 | 0.443 ± 0.018 | 0.424 ± 0.018 |
|  | Tuber/Root | 0.005 ± 0.000 | 0.082 ± 0.004 | 0.017 ± 0.000 |
| 10 | Culm/Root | 0.029 ± 0.002 | 0.499 ± 0.006 | 0.431 ± 0.042 |
|  | Tuber/Root | 0.007 ± 0.000 | 0.093 ± 0.007 | 0.029 ± 0.003 |

Table S2:TF values of Cu, Zn, and Cd in CES under combined metal stress. Note:Values are presented as mean ± SD (n = 3). BCF and TF values were calculated separately for each biological replicate and then averaged. BCF was calculated as the ratio of metal concentration in plant tissues to the corresponding metal concentration in the hydroponic solution. TF was calculated as the ratio of metal concentration in culms or tubers to that in roots. BCF and TF were not calculated for the control treatment because no metals were added to the nutrient solution.

**Supplementary Methods: Calculation of BCF and TF**

The bioconcentration factor (BCF) and translocation factor (TF) were calculated to evaluate the metal accumulation and translocation capacity of CES. BCF was calculated as the ratio of metal concentration in plant tissues to the corresponding metal concentration in the hydroponic solution:

$$BCF=C_{plant}/C_{solution}$$

where $C_{plant}$is the metal concentration in culms, roots, or tubers (mg/kg dry weight), and $C_{solution}$is the metal concentration in the nutrient solution (mg/L). TF was calculated as the ratio of metal concentration in culms or tubers to that in roots:

$$TF_{culm/root}=C_{culm}/C_{root}$$

$$TF_{tuber/root}=C_{tuber}/C_{root}$$

BCF and TF values were calculated separately for each biological replicate and then averaged. BCF and TF were not calculated for the control treatment because no metals were added to the nutrient solution.

|  | Accession numbers |
| --- | --- |
| MDA | A003-1-2 |
| H₂O₂ | A064-2-1 |
| Protein | A045-4-2 |
| Soluble sugar | A145-1-1 |
| Reducing sugar | A156-1-1 |
| Chlorophyll | A147-1-1 |
| Flavonoids | A142-1-1 |
| GSH | A006-2-1 |
| POD | A084-3-1 |
| SOD | A001-3-2 |

Table S3: Accession numbers of the commercial test kit.
